# Odontological Society of Pennsylvania

**Published:** 1894-10

**Authors:** 


### ODONTOLOGICAL SOCIETY OF PENNSYLVANIA.

The regular monthly meeting of the Odontological Society of Pennsylvania was held April 14, 1894, at 1228 Walnut Street, Philadelphia, the President, Dr. E. T. Darby, in the chair.

After the transaction of routine business the essayist of the evening, Dr. I. N. Broomell, read his paper on "Metallic *versus* Plastic Base for Artificial Dentures."

(For Dr. Broomell's paper, see page 620.)

### DISCUSSION.

*Dr. Guilford.*—The paper has proved a very interesting one, because it has dealt with what is commonly known as a practical subject. I was pleased to have it introduced, inasmuch as there are so many things connected with the making of plates that we do not understand. In what proportion of cases the metallic base is better, or the best, and in what proportion the vegetable, is hard to determine. While there are objections to the use of vulcanite, it certainly possesses some advantages. In my practice I have had a number of cases in which I did not succeed in getting a satisfactory fit with the metallic base, where I afterwards did with vulcanite; and, on the other hand, there have been cases where I have replaced vulcanite with gold with the very happiest results; so I think there is a large field for the employment of each.

It is deplorable, indeed, that with so good a material as vulcanite we should have so many ill results; it does seem as though every good thing in this world carried with it some objectionable features. Dentistry has suffered severely from the abuse of this material as it has also from painless extraction, amalgam, and bridge-work; all of them good and useful in their places, but followed by unfortunate results when improperly employed.

I think the abuse of vulcanite has come largely through careless manipulation and from the fact that we have not understood very well how best to manufacture it. Our knowledge in regard to the proper vulcanization of rubber for dental purposes falls very far short of that possessed by those who manufacture vulcanite for commercial and mechanical purposes.

When we try a new make of vulcanizable rubber we usually find a vulcanized sample in the box as an evidence of its quality; yet those pieces are generally vulcanized in the manner that rubber is vulcanized for mechanical purposes, between steel or iron dies and under the influence of steam at a higher temperature than we use. I think, in this matter of vulcanizing rubber, that many of the misfits are due to the fact that we do not understand how to manipulate the material. There are different theories in regard to the behavior of rubber during vulcanization. We understand some facts in regard to it, but we have not yet learned why in certain cases we cannot get a satisfactory fit with vulcanite, whereas in other cases the very best results are had under seemingly similar circumstances. Rubber, during the first stage of vulcanization, expands, and during the second stage contracts. Drs. Southwick and Snow both claim that in vulcanizing rubber it should be given a chance to expand while it is in a condition to do so, and afterwards it should be kept under very heavy pressure to prevent undue contraction. Dr. Southwick further believes that in the cooling of a plate the rubber contracts towards the part that is thickest, thus causing it to rise up and leave the model in the centre where it is thinner. For this reason the plate will rest upon the ridge, but not upon the palatal arch. He corrects this fault by reheating the plate and forcing it down in the centre after the flask has been opened.

I was told to-day by one who professed to know, in discussing this subject with him, that the large rubber rods which the electricians use, from an inch to an inch and a half in diameter, and as dense in the centre as at the surface, are vulcanized under steam at a high pressure and kept there for a long while, and that the steel moulds in which they are formed are kept under hydraulic pressure while undergoing the vulcanizing process. In this way only, he claimed, could solid and good results be obtained. We should take a hint from this and see whether we cannot improve our methods.

There are a great many points in regard to vulcanization under discussion and dispute, and it is a reproach to us that we do not understand them as thoroughly as we should. There are causes underlying the difficulties we are contending with, and I hope that in the near future we will get to the bottom of them.

In regard to the construction of metallic plates, I think success depends very largely upon our manipulation of the plaster in taking the impression and making the model, and afterwards in carefully and properly bringing the metal into uniform contact with the die in swaging.

The method of swaging described by Dr. Broomell in his paper is new, and seems to possess some valuable features. I first saw the description of it in the recent edition of Richardson's "Mechanical Dentistry," and I propose to give it a trial.

*Dr. Chupein.*—I would certainly recommend this plan of Dr. Broomell's in swaging a plate. I noticed it, as Dr. Guilford has said, in the late edition of Richardson's "Mechanical Dentistry." A plate, after it is swaged, ought to be exactly as the plate was before it was put on the die at all, only changed in form, and when it is at all stretched, as it is apt to be when driven by the horn hammer, the chances are that it will not fit at all. These successive counter-dies that are advocated by the essayist will effect this a great deal better than a horn hammer. Besides that, I am inclined to think that a plate brought up to the roof of the mouth in this way is not so much bruised as a plate would be if it was brought up by the use of the hammer.

I think there is an advantage in a gold plate over a rubber plate in certain mouths. Yet I have seen mouths that do not show what is called rubber-sore mouth, that kind of raw appearance, as frequently I have seen it under a gold plate; but I am very much inclined to think under both of these cases it is more due to uncleanliness than to anything else; because I have seen gums as healthy under a rubber plate—well taken care of—as I have seen the same under a gold plate. I think there is a great deal of truth in what Dr. Guilford has said in reference to the action of rubber, or as to the manner of vulcanizing it. There is a great deal to learn in the manner of vulcanizing. I have been perfectly nonplussed sometimes in trying to find out what is the cause of porosity in rubber, and have tried all kinds of experiments to determine the reason for this defect.

*Dr. Head.*—One can readily theorize concerning the tendency vulcanized rubber has to shrink towards the thickest part, if the vulcanizing is done on a plaster model, because the plaster is destroyed, thus leaving no means of attesting the accuracy of the deductions. But if the case is vulcanized on a tin model, it in each instance will accurately fit each indentation of the metal. This would seem to prove either that plaster has some evil effect on the rubber or that no such shrinkage takes place.

The success which attends the vulcanizing under high pressure would seem problematical. We dentists vulcanize under a pressure of eighty to one hundred pounds to the square inch, and frequently find our plates porous when the highest pressure is used. It is true that certain companies may vulcanize under a still higher pressure than any used in the dental profession, but it would not seem that pressure is the one panacea for porosity, as I know of a certain rubber shoe-factory where the vulcanizing is done at forty pounds, and yet the shoes are never porous.

In the light of these facts it would appear that the warping of plates, and their tendency to become porous, has yet to be satisfactorily explained.

In reference to the paper on swaging, I would say that Frank Faber told me of a method similar to this some ten years ago, although it was not perfected to such an extent as has been shown to-night. He *never* used the horn mallet, but simply bent the gold into the counter-die with his fingers, and drove it into shape by hammering on it with the zinc die. The only objection to this method was found in the fact that high arches tended to split the gold. This difficulty the lecturer this evening has beautifully obviated.

When Dr. Faber was swaging up a plate and wished to avoid cutting out the air-chamber, he used a case of zinc that did not run over the gums similar to the first counter-die spoken of by the lecturer this evening. With this case he drove the plate down around the air-chamber and into the interstices of the rugi.

This, of course, was exactly the same principle as the one set forth this evening, but we owe Dr. Broomell many thanks for the complete and scientific way in which he has developed it.

*Dr. Chupein.*—I forgot to mention when I was up that I had an idea some time ago of how vulcanite that had sprung could be brought back to its correct shape.

We all know that if we make a vulcanite clasp around a tooth, and the patient complains that it seems to be loose, by simply oiling the clasp and heating it gently with a spirit-lamp we can bend it with the pliers and change its shape so that it hugs the tooth tightly again. If you duplicate the model in any particular case, and make one of these on tin, you can bring up a whole plate that should happen to have sprung by pursuing the same plan. Put the plate on the die and make a case of plaster on the palatal surface of the plate; and by putting it in a flask press, and placing the case, press, and all in a stew-pan of oil, and heating the oil, you can so soften the rubber that the plate may be pressed up and made to fit the die accurately without the trouble of doing the whole work over with the chances of another misfit.

*Dr. Bonwill.*—Let us be liberal to one of our greatest aids for good,—the accused rubber plate.

Many who may not have used this article for plates have been very glad to make the so-called Coffin plate from this material.

How many of the graduates of to-day can make a respectable plate of any kind?

When in practice in Delaware, up to 1871, I had largely to deal with plate-work. I place in more gold than rubber, yet I am unwilling to concede the palm to the noble metal.

You are all aware that if gold were the only material endorsed by the societies, how very few of the poor would ever be able to wear artificial teeth.

While gold is the most valuable material for partial sets, yet rubber is not as bad as it is represented to be. I have used it for many years, and have yet to see the first effect of poisoning by the presence of mercury or through its non-conducting properties.

The subject was then passed, and the President called upon Dr. Rhone, of Bellefonte, formerly President of the Pennsylvania State Society, who had an obturator which he desired to exhibit.

(For Dr. Rhone's paper, see page 545.)

DISCUSSION.

*Dr. Guilford.*—Do you treat the soft palate in any way before taking the impression.

*Dr. Rhone.*—No.

*Dr. Guilford.*—No trouble?

*Dr. Rhone.*—No.

*Q.*—This bulb is solid?

*Dr. Rhone.*—Yes. My first experience was with a hollow bulb with a spring. That was unsatisfactory. I removed the spring, as the bulb was too light. I changed that for solid rubber, which has proved very satisfactory in this case. The bulb placed under in that way forces the instrument up in the upper parts and closes the nares entirely for the time being. The patient can breathe with it in place with the mouth closed comfortably. With the rigid obturator no part will move without moving the whole, and the difficulty she had was that the teeth, for instance, would suddenly move up and down. That, of course, is entirely obviated now, and she is perfectly comfortable.

*Dr. Deane.*—The subject of obturators is, in a measure, new to us as a society, but I have had some little experience with them. I find the most successful joint is the ball-and-socket joint, with which the parts to be restored can be thrown from side to side by the divided muscles of the soft palate while talking. It fills the space better and you secure better results. You do away more with the nasal twang. The rubber bulb, especially where you are trying to restore speech, can be handled easier and better than the uvula fastened with the hinge-joint.

I also find a great deal of benefit in those cases in using, first, soft rubber velum. This is made by vulcanizing two pieces or flaps of soft rubber together and attaching it to a plate with a pin-joint. You can move this up and down the rod or pin for the space of a quarter or half an inch until you find the proper point of articulation. This is a subject for experimental practice, and after your patient has worn an appliance of that kind through the experimental stages, you will find the general result will be much more successful when you put the hard rubber palate into the mouth.

My system of taking impressions differs a little from the Doctor's. I simply take an impression as for the ordinary hard-rubber plate, extending the rear portion back in the centre, in the form of a dovetail, and finish that plate up, adding any teeth that may be missing and banding teeth when possible; to this I would affix a bulb of impression compound, being careful to fasten it securely to the dovetail of the plate; this can then be kept warm and moulded to fit the open space in the cleft, both above and below the flap of divided tissue. I should think the Doctor would stand a chance of having severe trouble with the plaster impression shown here.

*Dr. Rhone.*—No; I have made it a point to have the patient bend forward and throw the head back, so that this could not possibly occur.

*Dr. Deane.*—That would eliminate the difficulty to a certain extent, but I should be very much afraid in those cases; they don't seem to have the ability to handle the muscles of the palate and throat, and such patients could not render any assistance at all. I came near having an accident, in a case of that kind, which led me to do away with impressions of the soft palate and adopt the modelling compound.

I have in mind a case of a child seven years of age which I treated with the soft rubber velum; the child is now twelve or thirteen years of age, and while the face and lips are disfigured he articulates well, and can sing and pronounce his words accurately, but after taking the plate out you can't understand a word he says. The experimental stage was the worst with soft rubber, but with hard rubber in his mouth he gets along very nicely.

*Dr. Guilford.*—In the few cases of cleft palate I have had I have found difficulty in taking the impressions, because the soft palate was so sensitive to the touch. I have generally found it necessary to treat the parts in some way before attempting to take the impression. Some operators recommend the application of camphor-water; others, manipulation with the fingers; but I have found that weak doses of bromide of potassium administered internally every three or four hours for a few times before the operation is sufficient to allay sensitiveness in cases of that character.

The plan spoken of by Dr. Rhone is very similar to that used by Dr. Baker, of Boston, in which he also uses the hard rubber bulb.

*Dr. Rhone.*—In regard to the hollow bulb, I experimented with that in this case first. I found it too light. It didn't drop when it should have dropped. This drops very nicely. There is no undue pressure on the mouth of this patient.

